# The X chromosome is necessary for ovule production in *Silene latifolia*

**DOI:** 10.1371/journal.pone.0217558

**Published:** 2019-05-23

**Authors:** Paris Veltsos, Lynda F. Delph

**Affiliations:** Department of Biology, Indiana University, Bloomington, Indiana, United States of America; United States Department of Agriculture, UNITED STATES

## Abstract

Sex chromosomes stop recombining and accumulate differences over time. In particular, genes on the chromosome restricted to the heterogametic sex degenerate and become non-functional. Here, we investigated whether or not the degeneration of a plant Y chromosome was sufficient to cause ovules containing a Y to fail to develop, thereby eliminating the possibility of YY individuals. We used two genotypic assays to determine the genotype—XX, XY, or YY—of offspring from a single fruit of an otherwise normal male XY plant of *Silene latifolia*. The fruit contained fewer ovules than normal pistillate flowers, produced an equal offspring sex ratio, and generated no YY offspring. The results indicate that ovaries must contain an X chromosome to develop properly. While haploid selection has slowed down Y-chromosome degeneration in *S*. *latifolia*, we find that it has progressed sufficiently to prevent the proper development of ovules, and hence prevent the presence of YY individuals.

## Introduction

Sex chromosomes evolve differently than the rest of the genome, because they are present in one sex more often than the other. The first plant sex chromosomes were identified in *Silene latifolia* almost a century ago [1,2], and have influenced early theoretical work on sex chromosomes. In fact, the canonical model of sex-chromosome evolution is based on plants, and describes the evolution of separate sexes from a hermaphrodite ancestor [3–5]. The model assumes the spread of a male-sterility mutation, resulting in a population of hermaphrodites and females. After the frequency of females increases, a second mutation—for female sterility—will spread in the population, as long as the resulting males can outcompete the existing hermaphrodites in male function, which is facilitated by existing inbreeding depression [3]. The presence of two separate sterility mutations, one for each sex, selects for a restriction of recombination between them, so that only male- and female-producing haplotypes occur, as they are the most fit possible haplotypes (the other possible haplotypes resulting in hermaphrodite, or completely sterile plants). Recombination is expected to gradually stop over more of the length of those proto-sex chromosomes, as they capture male- and female-beneficial mutations. This is because linkage with the sex-determining genes is advantageous for alleles with sexually antagonistic effects [6,7], i.e. alleles conferring a benefit to the fitness of one sex, while being deleterious for the other.

Characterization of the sex chromosomes of *S*. *latifolia* and closely related species has provided extensive evidence to support the canonical model of sex-chromosome evolution, and recent work in asparagus has demonstrated that 2 mutations are sufficient to turn autosomes into sex chromosomes [8]. The proto-sex chromosomes of *S*. *latifolia* stopped recombining 11 million years ago, and additional parts stopped recombining more recently, clearly defining evolutionary strata with different X-Y divergence estimates [9–11]. In addition, the non-recombining part of the Y has degenerated, as predicted by the canonical model [12,13]. Cytogenetic studies have demonstrated that the Y chromosome is larger than the X because of the accumulation of repeats such as transposable elements, organelle DNA and microsatellites [14–17]. Recent genomic analysis shows that about 50% of the genes that used to be shared between the X and the Y are now hemizygous on the X and that compensatory dosage compensation has evolved [10,11,18].

Some studies have investigated the viability of YY individuals, thus providing direct evidence of the fitness consequences of Y-chromosome degeneration. For example, indirect evidence comes from an inbred hermaphrodite plant carrying a mutant Y, which only produced female offspring when selfed or used as the male partner. This was interpreted as failure of gametophytes from this plant (pollen or ovules) containing a Y to support normal development [19]. More direct evidence comes from crosses of androhermaphrodite plants (with male and hermaphrodite flowers [20]). These plants were produced by disrupting the methylation profile of male plants, and as above, only female offspring were produced from these XY individuals, suggesting that the X chromosome is required to produce ovules. Lastly, recent androgenesis experiments, in which pollen grains were regenerated into mature plants, produced only XX individuals that were found to be diploidized [21]. The lack of YY individuals from androgenesis again suggests that the Y is insufficient for normal plant development.

A common caveat of studies that suggest that the Y lacks important genes for plant development is that they either involved mutant Y chromosomes or manipulations that do not occur in nature. Here we report offspring genotype ratios of seeds produced from a single fruit from an otherwise normal male plant, whose genotype was XY. Growing these seeds and determining their sex-chromosome genotype allowed us to determine whether an X chromosome is required for normal ovule development. If ovules containing Y chromosomes existed and were viable, and if no other mechanisms were at work, an offspring genotype ratio of 1XX:2XY:1YY would be expected. If Y ovules were produced but YY offspring were inviable, we would expect a 1XX:2XY ratio. Lastly, if ovules containing Y chromosomes did not exist or were inviable, the resulting ratio would be 1XX:1XY. We found no YY offspring and an equal sex ratio in the F_1_ offspring.

## Materials and methods

The plant used in this study came from a cross between individuals from a population in Virginia (37°21'36''N, 80°40'54''W, 500 m above sea level). The study was carried out on private land, the owner gave permission to conduct the study on this site and the study did not involve endangered or protected species. The natural field population had a sex ratio of 54.5% females (697 females, 582 males) in 2018. The seed was germinated in a greenhouse at Indiana University, and following a 2-week growth period in the greenhouse, the resulting seedling was transplanted into a 2L pot that was placed outside in Bloomington, IN for a 3-month period (from 30 May to 30 Aug 2018). The focal plant produced 1150 normal staminate (pollen-producing) flowers during its 85 days of flowering, and 1 pistillate (ovule-producing) flower that developed into a fruit. This individual was otherwise indistinguishable from a normal male. For example, the mean calyx width (a very sexually dimorphic character) of other males from this population used in this planting was 7.33 (± 0.14 SE) and the mean calyx width for this plant was 7.85. In contrast, females from this planting had calyces that averaged 9.35 (± 0.19 SE). The focal plant was surrounded by other *S*. *latifolia* plants, allowing the flower that produced the fruit to be open pollinated. Hence, all offspring from the fruit are expected to be the product of a XY x XY cross, which would produce a genotype ratio of 1 XX: 2 XY: 1YY if Y ovules were produced and YY individuals were viable. The chisquare tests were conducted in R v 3.5.3 [22] using the chisq function.

**Fig 1:**
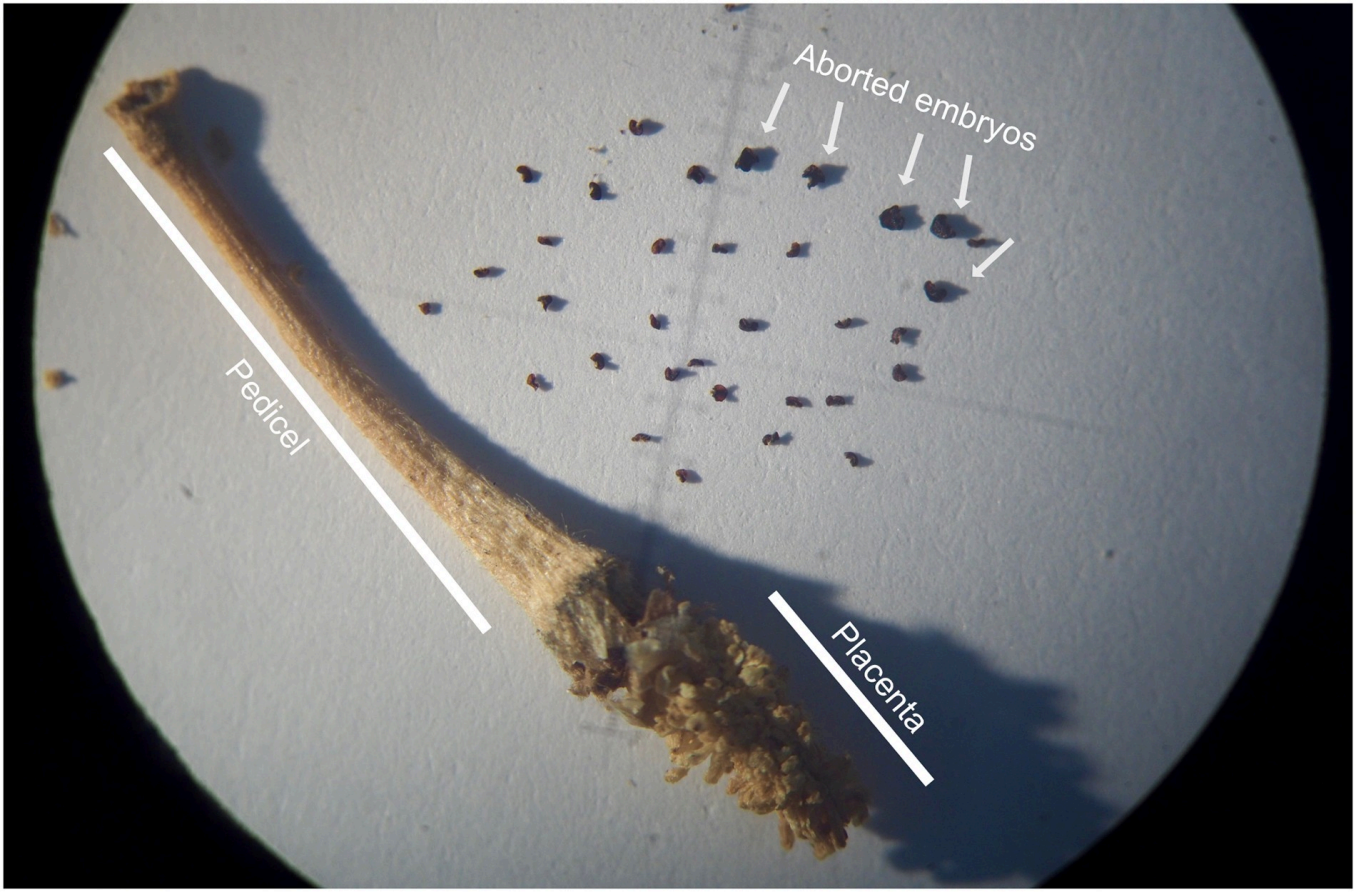
The mature fruit of the XY individual with the calyx, ovary wall, and seeds removed. The pedicel and placenta, both maternally derived tissues, had their DNA extracted. There were 27 non-fertilized ovules and 5 aborted embryos (arrows).

All mature seeds from the fruit were planted in a 50:50 Metromix (Scotts-Sierra Horticultural Products, Marysville, OH):greenhouse soil mixture, and transplanted to 1L pots at the 4-leaf stage. The plants were kept at ~24°C in 16h:8h light:dark cycle to induce flowering. The plants that had flowered by the time of DNA extraction were used as controls for the PCR assays, and by the end of the experiment all but 2 of 66 had flowered. We also performed the genotyping assays on DNA extracted from the naturally dried pedicel and placental tissue of the fruit of the XY individual (Fig 1) as positive controls in the PCR. Young leaves from all male offspring, and offspring that had not flowered, were processed for DNA extraction within 2 hours of collection. Each sample was frozen in liquid N_2_ and ground to a powder, in mortars and pestles that had been kept at -20°C. The ground plant material was processed with a DNeasy Plant Mini kit (Qiagen) according to the manufacturer’s instructions.

**Fig 2:**
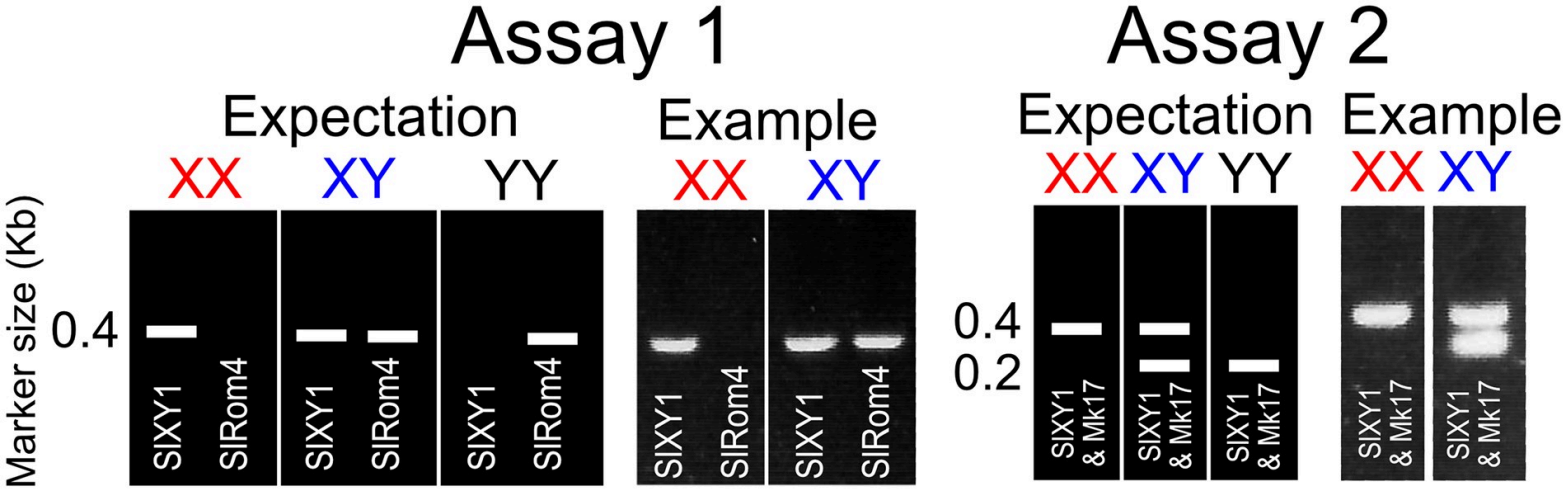
Expected results from the two assays used to determine whether the plants contained X and/or Y chromosomes. An example gel picture is illustrated next to each expectation.

All PCR reactions were in a 20 μl reaction volume and used HotStarTaq Master Mix Kit (Qiagen) with final primer concentration 2 μM and 1.5 mM MgCl_2_. To identify whether a plant contained X and/or Y chromosomes, we performed two genetic assays. Assay 1 used primers SL4RomF (GAGACACCGACAACTGTCCA) and Sl4RomR (ACGCAAAATACAGCGACACA), which reliably amplify a Y-specific 400 bp band across many *S*. *latifolia* populations (R. Hobza, personal communication). Some females also amplified higher molecular weight products with the Sl4RomR and Sl4RomF primers which were never seen in males (S1 Fig). To confirm the genotyping, we performed Assay 2, which used primer pairs MK17F, MK17R and SLXY1F, SLXY1R together in the same PCR reaction [23]. Two Y-specific bands were expected to be produced from the SLXY1 primer pair (at ≈200 bp and ≈1000 bp) along with a X-specific band at 400 bp [23]. However only the 200 bp band was observed (Fig 2 and S1 Fig), even after increasing the PCR extension time to 90 s, suggesting it is not present in our population. The expected PCR band pattern for the two assays is shown in Fig 2 and results for all individuals are summarized in S1 Fig. The PCR program for all primers was 95°C for 15 min, followed by 35 cycles of 95°C 30s, 55°C at 45 s, 72°C for 45 s and final extension at 72°C for 5 min.

## Results

### Fruit characterization

The flower from the male plant that produced the fruit contained 112 ovules—an uncharacteristically small number as the average for this population is ~300 (unpublished data/L. Delph personal communication). The pedicel of the fruit had an XY genotype, as did the placental tissue. The fruit contained 27 non-fertilized ovules, 5 aborted embryos, and 80 seeds (Fig 1). Of these 80 seeds, 66 germinated, which is a typical germination rate. The majority of fertilized ovules therefore matured normally.

### Plant genotyping by PCR—Absence of YY offspring in the F_1_

The inferred genotype of the plants agreed with their observed sex for all 64 plants that had flowered by the end of the experiment. Eight of these plants were not genotyped as they were females, and the molecular assay was aimed at differentiating males with a XY or YY genotype. Only 2 plants had not flowered and their sex was inferred only through genotyping. All flowers of both male and female offspring were normal.

There were 38 XX, 28 XY and no YY offspring. This deviates from the 1XX:2XY:1YY ratio (X^2^ = 45.3, df = 2, *p* < 0.001) expected if ovules containing a Y chromosome were produced and YY offspring were viable. Four observations combined indicate that Y ovules failed to be produced: the low number of ovules produced by the flower, the low number of fertilized embryos that were aborted, the absence of YY offspring, and an offspring genotype ratio that deviated significantly from a 1XX:2XY ratio (X^2^ = 17.5, df = 1, *p* < 0.001). The expectation based on these findings is a 1XX:1XY offspring ratio. While there were more females than males in the offspring (58% female-biased), the sex ratio did not significantly differ from 50% (X^2^ = 1.5, df = 1, *p* = 0.22), or from the 54.5% female-biased ratio of the parental population (X^2^ = 0.25, df = 1, p = 0.62; note that the parental population sex ratio might be affected by differential survival for the two sexes).

## Discussion

To test whether an X chromosome is required to produce viable seed in the dioecious species *S*. *latifolia*, which has heteromorphic sex chromosomes, we took advantage of seeds produced from a single ovule-bearing flower from a plant that was otherwise a normal XY male individual. The complete absence of YY offspring from the fruit that developed from this flower suggests that the X contains genes not present on the Y that are necessary for megasporophyte development, such that no ovules with a Y develop. The lack of development of ovules containing a Y is further supported by the fact that the XY fruit contained abnormally few ovules.

One possible cause of deleterious effects on the megasporophyte is the Y chromosome itself, since about half of its genes have degenerated [10,11,24]. Indirect effects involving the Y chromosome are also possible, such as interactions between cytoplasmic sex-ratio distorters and Y-chromosome restorers that are thought to produce the female-biased sex ratios characteristic of many natural populations of *S*. *latifolia* [25]. The same deleterious interactions may result in developmental failure before zygote formation in XY fruit, as they are likely to disproportionately affect tissues with high metabolic needs.

Another mechanism generating a deficit of ovules with a Y chromosome is meiotic drive, which is the non-Mendelian inheritance of chromosomes [26] and is a common phenomenon. For example, it can come about by variation in the centromeres of chromosome pairs which can bias some non-sister chromatids into transmitting to the egg and avoiding the polar body. This has been found in monkeyflowers [27]. Y chromosomes have not evolved in a meiotic environment where they need to compete for such transmission, since they normally do not pass through female meiosis. In contrast, an arms-race competition for transmitting to the egg, among X-chromosome variants, might have resulted in efficient transmission of the X into the egg, resulting in a less likely transmission of the Y chromosome to the egg when it competes with a X chromosome, in rarely occurring XY fruit. Nevertheless, meiotic drive cannot fully account for our data, since it is expected to bias the sex chromosome reaching the ovaries to be the X, rather than reduce the number of mature ovaries; i.e. it cannot account for the failure of ovules containing a Y chromosome to develop. Moreover the Y chromosome transmits through females in XXYY tetraploid *S*. *latifolia* hermaphrodites [28], making meiotic drive an unlikely explanation for our data.

In addition to excluding meiotic drive as the underlying cause of our results, we were also able to exclude spontaneous loss of the Y chromosome to the part of the plant that produced the female flower. We confirmed its XY genotype by directly genotyping the pedicel and placental tissue (S1 Fig). We can also exclude major post-fertilisation YY inviability effects, because only 5 fertilized ovules aborted during development (Fig 1). We cannot exclude the possibility that the low ovule number observed may be a consequence of the smaller size of the flower meristem in males [29], and that the Y chromosome need not be solely responsible for it.

Our results contrast those from Miller & Kesseli (2001)[30] where several XY plants were shown to transmit the Y through ovules and a ratio of 1XX:2XY was obtained. This is likely a consequence of the naturally occurring Y studied by Miller & Kesseli having a mutation that disrupts its female suppression function (see [31]). This would make it similar to the Y_2_ described by Westergaard (1946)[28], although smaller mutations that do not affect the chromosome structure can have the same effect. Such a disruption would result in the observed hermaphrodite phenotype of the XY individuals used in their experiment, and the complete absence of normal males. In contrast to previous studies of Y-chromosome phenotypic effects in *S*. *latifolia*, we have no reason to believe the Y chromosome in our study is different from wild type. Only one out of 1151 flowers was pistillate in the parent plant, and none of the F_1_ male offspring had abnormal flowers. The reason the Y chromosome studied by Miller & Kesseli could pass through ovaries is either because the missing female suppressor function gene on that chromosome pleiotropically affects ovule viability, or because a larger translocation of X or autosomal genes had occurred (as in Westergaard’s Y_2_[28]) that restored some of the lost functionality of the wild-type Y.

Taken together, our results provide the first evidence of negative fitness consequences of Y-chromosome degeneration using a naturally occurring Y chromosome in an otherwise normal male in *S*. *latifolia*. Our study adds to the body of evidence that YY plants are inviable based on artificial means (e.g., mutant Y chromosomes and disruption of methylation [1,20,21]). The departure from a 1:2 XX:XY sex ratio in the offspring and the low number of total ovules in our study allows us to conclude that the development of the female gametophyte requires an X chromosome. In addition, the small number of ovules contained in the fruit excludes meiotic drive as an explanation for the deficit of XY individuals. While haploid selection slows down Y-chromosome degeneration [11,32], we conclude that the Y chromosome of *S*. *latifolia* has degenerated to the extent that it is insufficient for ovule development.

## Supporting information

S1 Fig Sample genotyping. Eight female offspring that flowered before DNA collection were not genotyped. The DNA ladders used were either the 100 bp Promega ladder or NEB HindIII λ ladder. The inferred genotypes are indicated with colored text when they agreed with the phenotypic sex of the offspring. Samples where the genotype did not agree with the phenotypic sex, are annotated with the symbol of the phenotypic sex and were genotyped again using both genotyping assays, along with samples where PCR failed (bottom). Some female samples (3, 4, 10, 19, 22, 28, 31, 41, 45) amplified higher molecular weight bands in an inconsistent fashion (e.g. sample 41 repeated within the same assay (top) and samples 19, 28, 31, which only showed the band in the top assay).
(TIF)
