# Supplementary figures and images for: The X chromosome is necessary for ovule production in Silene latifolia
Source: PLoS One. 2019 May 23;14(5):e0217558. doi: 10.1371/journal.pone.0217558 (PMC6533010; doi:10.1371/journal.pone.0217558)

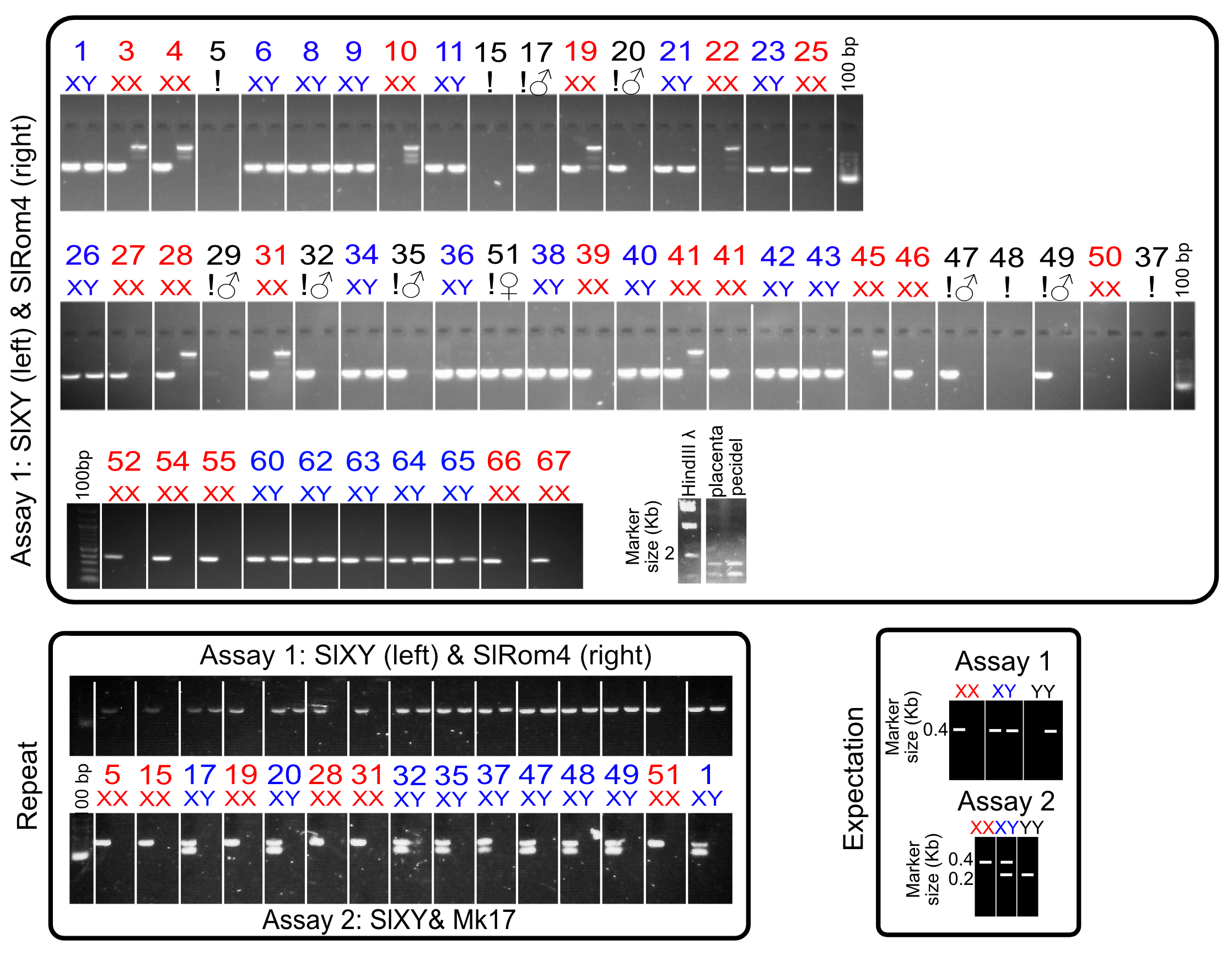

Supplement: S1 Fig — Eight female offspring that flowered before DNA collection were not genotyped. The DNA ladders used were either the 100 bp Promega ladder or NEB HindIII λ ladder. The inferred genotypes are indicated with colored text when they agreed with the phenotypic sex of the offspring. Samples where the genotype did not agree with the phenotypic sex, are annotated with the symbol of the phenotypic sex and were genotyped again using both genotyping assays, along with samples where PCR failed (bottom). Some female samples (3, 4, 10, 19, 22, 28, 31, 41, 45) amplified higher molecular weight bands in an inconsistent fashion (e.g. sample 41 repeated within the same assay (top) and samples 19, 28, 31, which only showed the band in the top assay). (TIF) [file pone.0217558.s001.tif]
